# MiRNA-target network analysis identifies potential biomarkers for Traditional Chinese Medicine (TCM) syndrome development evaluation in hepatitis B caused liver cirrhosis

**DOI:** 10.1038/s41598-017-11351-5

**Published:** 2017-09-08

**Authors:** Yamin Liu, Mei Wang, Yunquan luo, Jian Chen, Yiyu lu, Yulin Shi, Chenchen Tang, Qianmei Zhou, Hui Zhang, Yuanjia Hu, Shibing Su, Qilong Chen

**Affiliations:** 1 Research Center for TCM Complexity System, Shanghai University of TCM, Shanghai, 201203 China; 2 School of Clinical Medicine, Shanghai University of Medicine & Health Sciences, Shanghai, 201203 China; 3 Shu Guang Hospital Affiliated to Shanghai University of TCM, Shanghai, 201203 China; 4 State Key Laboratory of Quality Research in Chinese Medicine, Institute of Chinese Medical Sciences, University of Macau, Macau, China

**Keywords:** Gene expression, Hepatitis B

## Abstract

Hepatitis B is one of most etiologies of Liver cirrhosis in China, and clinically lacks the effective strategy for Hepatitis B caused cirrhosis (HBC) therapy. As a complementary and alternative medicine, Chinese Traditional Medicine (TCM) has special therapeutic effects for HBC. Here, we focus on the evolution process of HBC TCM syndromes, which was from Excessive (Liver-Gallbladder Dampness-Heat Syndrome, LGDHS) to Deficient (Liver-Kidney Deficiency Syndrome, LKYDS) via Excessive-Deficient syndrome (Liver-Depression and Spleen-Deficiency Syndrome, LDSDS). Using R package, 16 miRNAs in LGDHS/Normal, 48 miRNAs in LDSDS/LGDHS, and 16 miRNAs in LKYDS/LDSDS were identified, respectively. The miRNA-target networks show that the LDSDS was most stability and complicated. Subsequently, 4 kernel miRNAs with LGDHS-LDSDS process, and 5 kernel miRNAs with LDSDS-LKYDS process were screened. Using RT-qPCR data, p1 (hsa-miR-17-3p, -377-3p, -410-3p and -495) and p2 miRNA panel (hsa-miR-377-3p, -410-3p, -27a-3p, 149-5p and 940) were identified by Logistic Regression Model, which clearly improve the accuracy of TCM syndrome classification. The rebuilt miRNA-target network shows that the LDSDS is a critical point and might determine the evolution directions of HBC TCM syndrome. This study suggests that the identified kernel miRNAs act as potential biomarkers and benefit to evaluate the evolution tendency of HBC TCM syndromes.

## Introduction

Liver Cirrhosis has been recognized by the development of acute deterioration of liver function^1^. Importantly, hepatitis B Virus (HBV) caused cirrhosis (HBC) is one of most etiologies of Liver cirrhosis and annually about 1.5 million people are suffering in China^2^. As a chronic and complicated liver disease, the 5-year survival rate of patients with severe HBC only is about 50%^3^, especially, hepatocellular carcinoma (HCC) was estimated that occurs almost exclusively in patients with HBC^4^. During the past decades, clinically lacks the effective strategy for the therapy of HBC, fortunately, as a complementary and alternative medicine, Chinese Traditional Medicine (TCM) has special therapeutic effects for HBC treatment^5^. In previous study, we found that PNP, AQP7, and PSMD2 may be involved in TCM syndromes differentiation of HBC^6^. However, the characteristics of HBC TCM syndromes are unclear, especially, the development mechanism from Excessive (Liver-Gallbladder Dampness Heat Syndrome, LGDHS) to Excessive-Deficient (Liver-Depression and Spleen-Deficiency Syndrome, LDSDS) syndrome, and/or Excessive-Deficient (Liver Depression and Spleen Deficiency Syndrome, LDSDS) develop to Deficient (Liver-Kidney Deficiency Syndrome, LKYDS) syndrome still rigorously lacks in HBC progression.

MicroRNA (miRNA) is a class of small nonconding RNA molecules (18–24 nucleotides), which was involved in many liver disease, including liver metabolism, fibrosis, regeneration, and HCC^7, 8^. Importantly, the exceptional stability of miRNA in serum or plasma is valued for clinical use, such as playing biomarkers to distinguish chronic HBV hepatitis, their corresponding cirrhosis and HCC^9, 10^. In this work, we analyzed the dynamical expression levels of miRNAs in the evolutionary process of HBC TCM syndromes based on transcriptional profiles. The miRNA-target network suggests that the LDSDS might act as an important critical point in HBC TCM syndromes development. Using the RT-qPCR data, we have established stepwise logistic regression models with promising diagnostic performances for TCM syndrome classification. Our findings provide useful information for developing novel tools to evaluate the evolution tendency of HBC TCM syndromes.

## Results

### Differential expressed miRNAs of TCM syndromes in HBC

**Figure 1:**
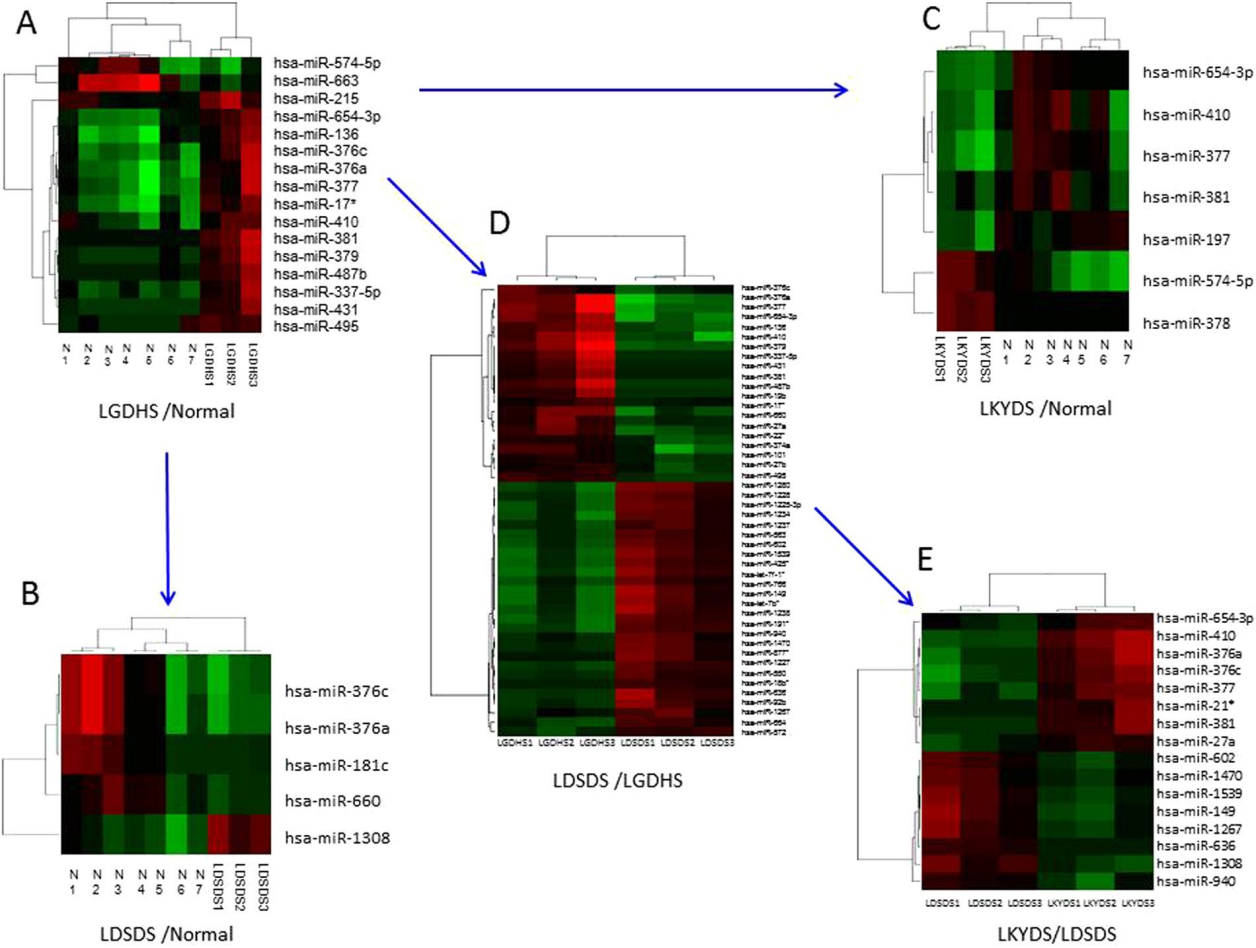
Hierarchical cluster and heat map of HBC TCM syndromes. (**A**) represents the differential expressed miRNAs between LGDHS and Normal, (**B**) represents the differential expressed miRNAs between LDSDS and Normal, (**C**) represents the differential expressed miRNAs between LKYDS and Normal, (**D**) represents the differential expressed miRNAs between LGDHS and LDSDS, and (**E**) represents the differential expressed miRNAs between LDSDS and LKYDS.

Using the Random variance model of R package, 16 differential expressed (DE) miRNAs in LGDHS/Normal, 5 DE miRNAs in LDSDS/Normal, and 7 DE miRNAs in LKYDS/Normal were identified, respectively (Fig. 1A–C). It suggests that the expression statuses of miRNAs are great difference among the three HBC TCM syndromes. In this study, we mainly focus on the miRNAs levels among the LGDHS/Normal, LDSDS/LGDHS, and LKYDS/LDSDS, because they represent a consecutive evolutionary stage of HBC TCM syndromes, which was evolved from Excessive syndrome (LGDHS) to Deficient (LKYDS) via Excessive-Deficient (LDSDS) syndrome. Subsequently, 48 DE miRNAs in LDSDS/LGDHS (Fig. 1D) and 16 DE miRNAs in LKYDS/LDSDS (Fig. 1E) were obtained, respectively, and suggesting that these DE miRNAs are high correlated with the process of LGDHS developed to LDSDS and LDSDS evolved to LKYDS. Furthermore, 6 overlapped miRNAs, including hsa-miR-376a-3p, -376c-3p, -377-3p, -381-3p, -410-3p, and -654-3p, were selected from the LGDHS/Normal, LDSDS/LGDHS, and LKYDS/LDSDS and defined as co-expressed miRNAs in the evolutionary process of TCM syndromes, suggesting these miRNAs maybe play critical roles for HBC development.

**Figure 2:**
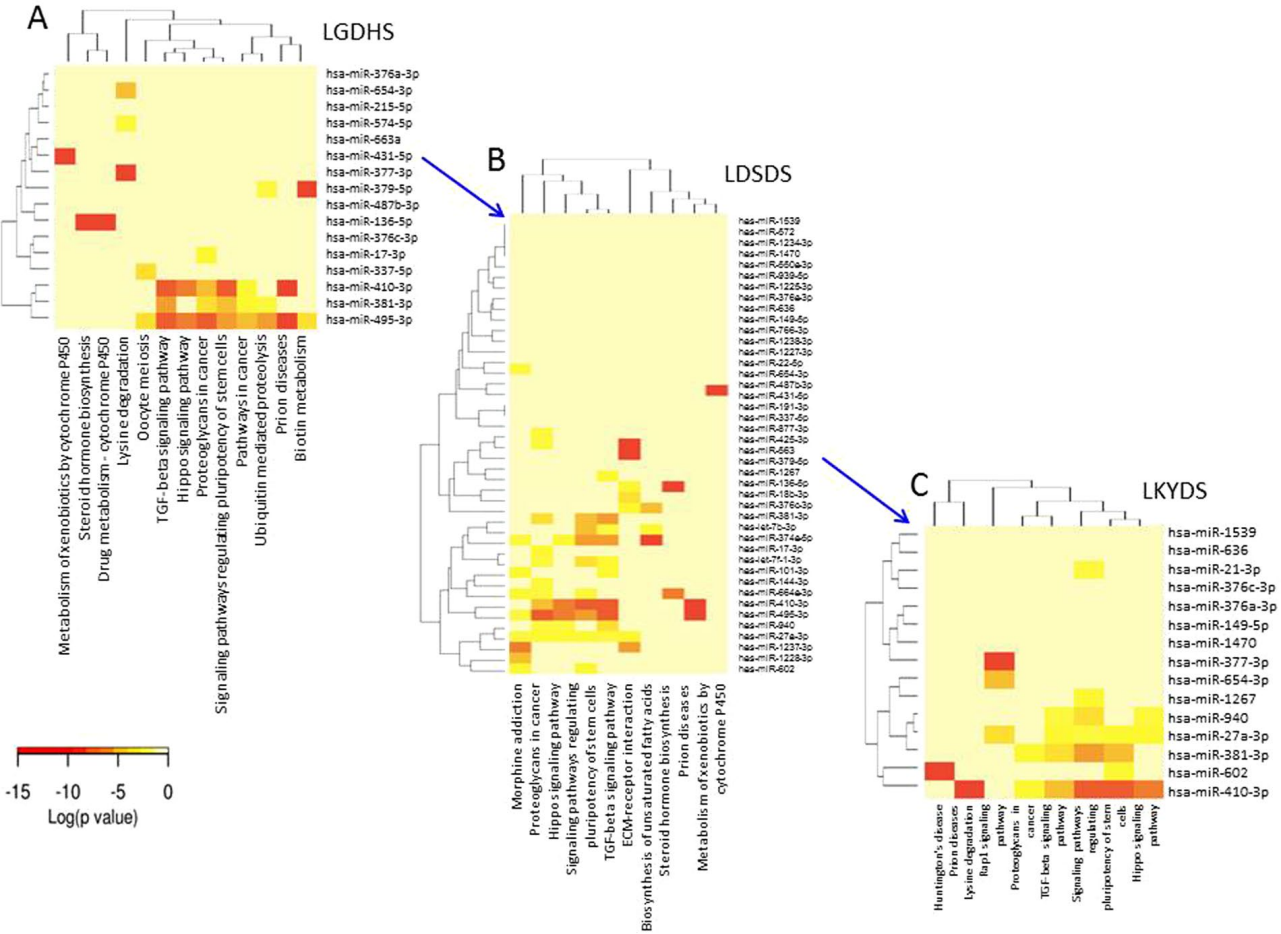
The hierarchical cluster and heat map of miRNAs versus KEGG pathways were calculated using their interaction levels in the evolutionary process of HBC TCM syndromes, (**A**) represents the LGDHS, (**B**) represents the LDSDS, and (**C**) represents the LKYDS. The pathway analysis were performed using miRPath program, and the significant pathways were determined when P-value < 0.001.

To investigate the biological functions of the DE miRNAs in evolutionary process of HBC TCM syndromes, the miRPath (v3.0) program were performed for pathways analysis^11^, and significant terms were determined when *P*-value < 0.001. Compare with the normal controls, Steroid hormone biosynthesis, Prion diseases, Metabolism of xenobiotics by cytochrome P450, TGF-beta signaling pathway, Signaling pathways regulating pluripotency of stem cells, Proteoglycans in cancer, Lysine degradation, and Hippo signaling pathway are highly associated with LGDHS (Fig. 2A). In the process of LGDHS developed to LDSDS, the pathways mainly focus on ECM-receptor interaction, TGF-beta signaling pathway, Signaling pathways regulating pluripotency of stem cells, Steroid hormone biosynthesis, Proteoglycans in cancer, and Prion diseases (Fig. 2B). Furthermore, the TGF-beta signaling pathway, Prion diseases, Signaling pathways regulating pluripotency of stem cells, Lysine degradation, and Huntington’s disease are highly correlated with the process of LDSDS develop to LKYDS (Fig. 2C). Interestingly, three pathways thread throughout the whole evolutionary process of LGDHS-LDSDS-LKYDS, such as Prion diseases, Signaling pathways regulating pluripotency of stem cells, and TGF-beta signaling pathway. It suggests that these pathways are indispensable for the evolutionary process of HBC TCM syndromes.

### miRNA target genes prediction and enrichment analysis

**Figure 3:**
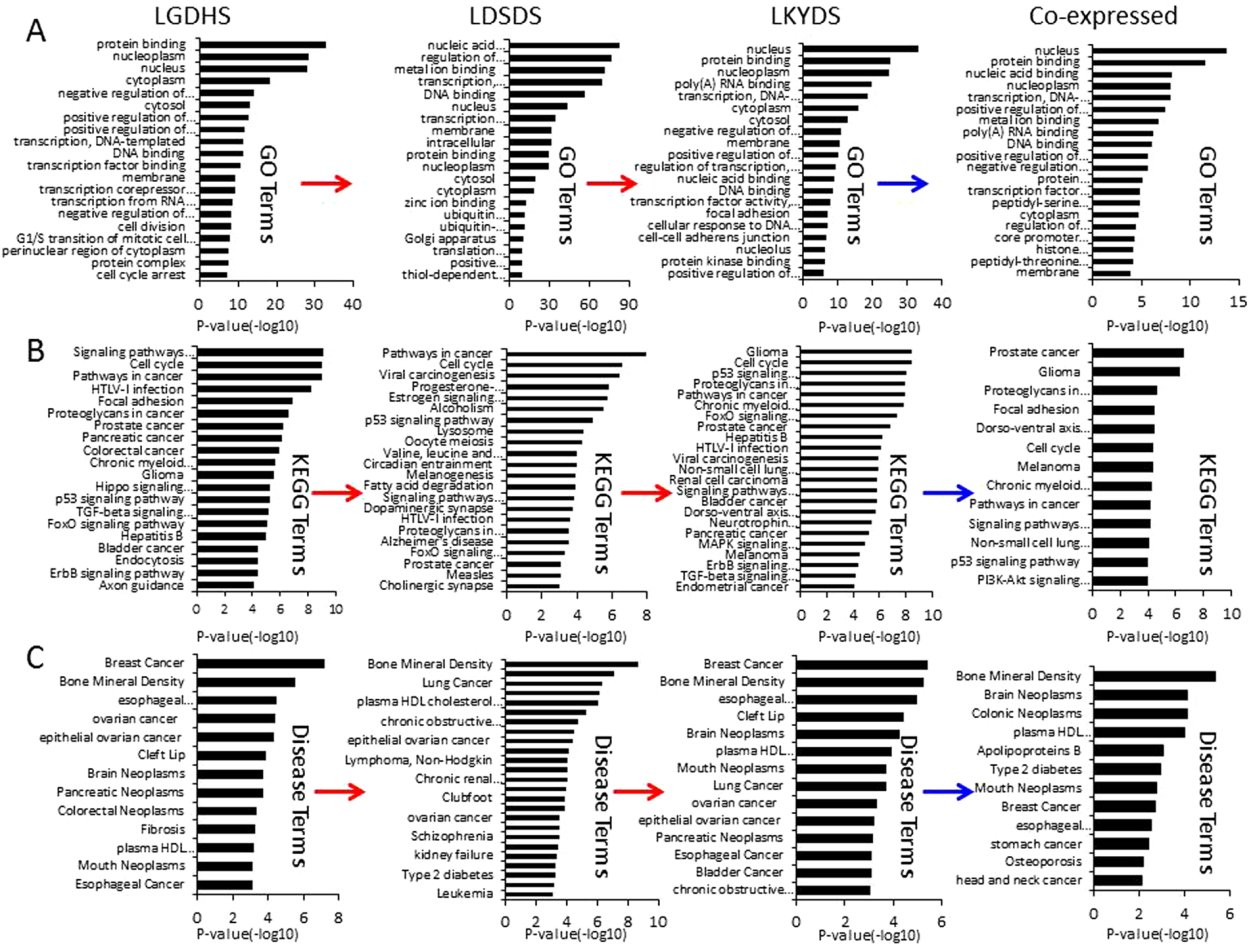
The biological functions analysis of differential expressed miRNAs target genes. (**A**) Represent the GO terms distributions in the HBC TCM syndrome development, (**B**) represent the KEGG pathways distributions in the HBC TCM syndrome development, and (**C**) represent the Disease terms distributions in the HBC TCM syndrome development.

Using the prediction programs, the miRNA target genes were predicted respectively. After redundancy analysis, the final database of LGDHS/Normal (16 DE miRNAs with 2917 target genes), LDSDS/LGDHS (48 DE miRNAs with 6531 target genes) and LKYDS/LDSDS (16 DE miRNAs with 2594 target genes) were built, respectively. The functional enrichment was conducted by DAVID program, GO terms (10% top terms), KEGG pathways and Disease terms were shown in Fig. 3. In addition to the same GO, KEGG and Disease terms, the LGDHS related biological functions mainly associated with regulation of transcription, cell division, G1/S transition of mitotic cell cycle, cell cycle arrest, Focal adhesion, Pancreatic cancer, Chronic myeloid leukemia, Glioma, Hippo signaling pathway, TGF-beta signaling pathway, Hepatitis B, and ErbB signaling pathway. The LDSDS mainly associated with metal ion binding, transcription factor activity, ubiquitin conjugating enzyme activity, ubiquitin-dependent protein catabolic process, Viral carcinogenesis, Progesterone-mediated oocyte maturation, Estrogen signaling pathway, leucine and isoleucine degradation, Melanogenesis, Fatty acid degradation, Dopaminergic synapse, and Alzheimer’s disease. The LKYDS mainly associated with poly (A) RNA binding, cellular response to DNA damage stimulus, cell-cell adherens junction, protein kinase binding, Hepatitis B, Dorso-ventral axis formation, Neurotrophin signaling pathway, MAPK signaling pathway, ErbB signaling pathway, and many tumors. These results suggested that the biological functions are great difference among the three HBC TCM syndromes.

Here, we notice that the co-expressed miRNAs thread throughout the whole evolutionary process of HBC TCM syndrome, which might play important roles for TCM syndrome evolution. GO terms (*P* < 10^−5^) show that these co-expressed miRNAs target genes are mainly associate with nucleus, protein binding, nucleic acid binding, nucleoplasm, transcription, metal ion binding, poly(A) RNA and DNA binding, protein serine/threonine kinase activity, and transcription factor activity (Fig. 3A). KEGG pathways show an impressive functional association with various signal-related and cancer-related pathways, such as Signaling pathways regulating pluripotency of stem cells (*P* = 6.78 × 10^−5^), p53 signaling pathway (*P* = 1.07 × 10^−4^), and PI3K-Akt signaling pathway (*P* = 1.02 × 10^−4^), Prostate cancer (2.67 × 10^−7^), Glioma (5.22 × 10^−7^), Proteoglycans in cancer (2.09 × 10^−5^), Pathways in cancer (6.62 × 10^−5^), Non-small cell lung cancer (8.70 × 10^−5^) (Fig. 3B). Furthermore, Disease terms show that the co-expressed miRNAs target genes mainly associated with Bone Mineral Density, Brain Neoplasms, Colonic Neoplasms, plasma HDL cholesterol levels, Apolipoproteins B, Type 2 diabetes, Mouth Neoplasms, Breast Cancer, esophageal adenocarcinoma, stomach cancer, Osteoporosis, head and neck cancer (Fig. 3C). Although none of the Disease terms directly correlated with hepatitis B or fibrosis, in fact, many terms belong to the complicated disease and may be relevant to the genesis of liver cirrhosis.

### miRNA-target networks building and kernel miRNAs screening

**Figure 4:**
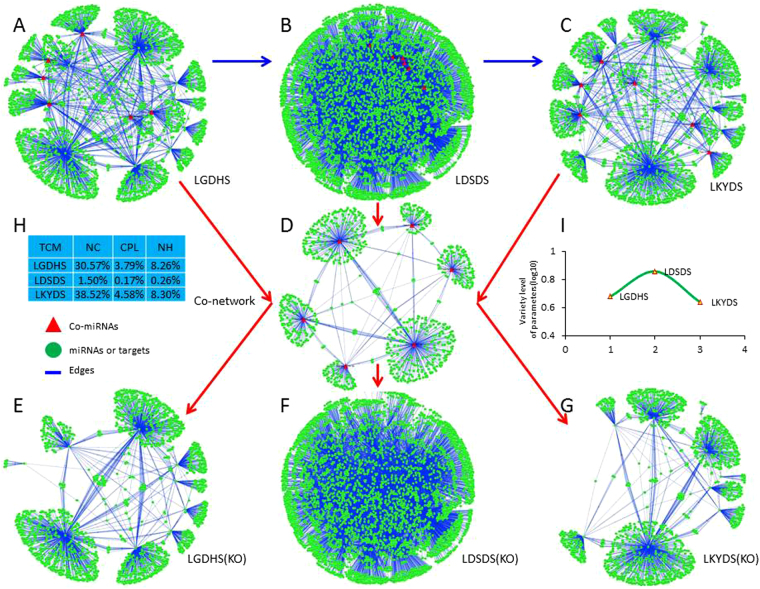
The miRNA-target network construction in the in the HBC TCM syndrome development. (**A**) Represents the global profiles of LGDHS network, (**B**) represents the global profiles of LDSDS network, (**C**) represents the global profiles of LKYDS network, and (**D**) represents the co-network. To investigate the functions of co-expressed miRNAs, the co-network was knockout form the three miRNA-target networks, respectively, (**E**) represents the LGDHS knockout network, (**F**) represents the LDSDS knockout network, and (**G**) represents the LKYDS knockout network. (**H**) Represents the change rate of network parameters between before and after knockout, and (**I**) represents the Robustness levels in three networks.

In the evolution process of HBC TCM syndromes, the miRNA-target networks of LGDHS, LDSDS and LKYDS (Fig. 4A–C) were constructed, respectively. The topological profiles show that three networks are more likely similar to ‘Medusa’ model^12^, which consists of regulatory core framework by hub nodes and represent most determinants in the network. This model suggests that the hub nodes have prominently functions in the realized network profiles, and the periphery nodes only should be regulated^13^. Such being the case, the network parameters were calculated, including Betweenness Centrality (BC), Closeness Centrality (CC) and Degree (De), and the hub nodes of networks were defined as BC ≥ Avg (BC), CC ≥ Avg (CC) and De ≥ Avg (De). The determinate nodes were considered as kernel miRNAs and play important roles for TCM syndrome evolution in HBC progression. Finally, 7 kernel miRNAs in LGDHS network, 17 kernel miRNAs in LDSDS network, and 8 kernel miRNAs in LKYDS network were screened, respectively (Supplementary Table 1). Furthermore, we noticed 4 kernel miRNAs shared by LGDHS and LDSDS, 5 kernel miRNAs overlapped between LDSDS and LKYDS, and 2 kernel miRNAs (hsa-miR-377-3p, -410-3p) mutually shared among the LGDHS, LDSDS and LKYDS. The results indicated that these kernel miRNAs might directly regulate the Excessive (LGDHS) develop to Excessive-deficient syndrome (LDSDS) or Excessive-deficient (LDSDS) develop to Deficient syndrome (LKYDS).

### The co-network was knockout from the miRNA-target network

Furthermore, the co-network of three TCM syndromes was isolated from above networks (Fig. 4D). To investigate the functions of these co-expressed miRNAs, the co-network was knockout from three networks, respectively (Fig. 4E–G), subsequently, the network parameters between before and after knockout were calculated, such as Network centralization (NC), Characteristic path length (CPL), and network heterogeneity (NH). In the LGDHS and LKYDS networks, the variable ratios of NCs are 30.57% and 38.52%, CPLs are 3.79% and 4.58%, and NHs are 8.26% and 8.30%, whereas, the related parameters of LDSDS network are only 1.50%, 0.17%, and 0.26%, respectively (Fig. 4H). Furthermore, the Robustness levels also demonstrated the LDSDS network (R = 0.87) is more stability than LGDHS(R = 0.68) and LKYDS networks (R = 0.64) (Fig. 4I). The results suggested that these co-expressed miRNAs might more important for LGDHS and LKYDS syndromes in the evolutionary process of HBC TCM syndromes.

### Validating the co-expressed miRNAs and kernel miRNAs

**Figure 5:**
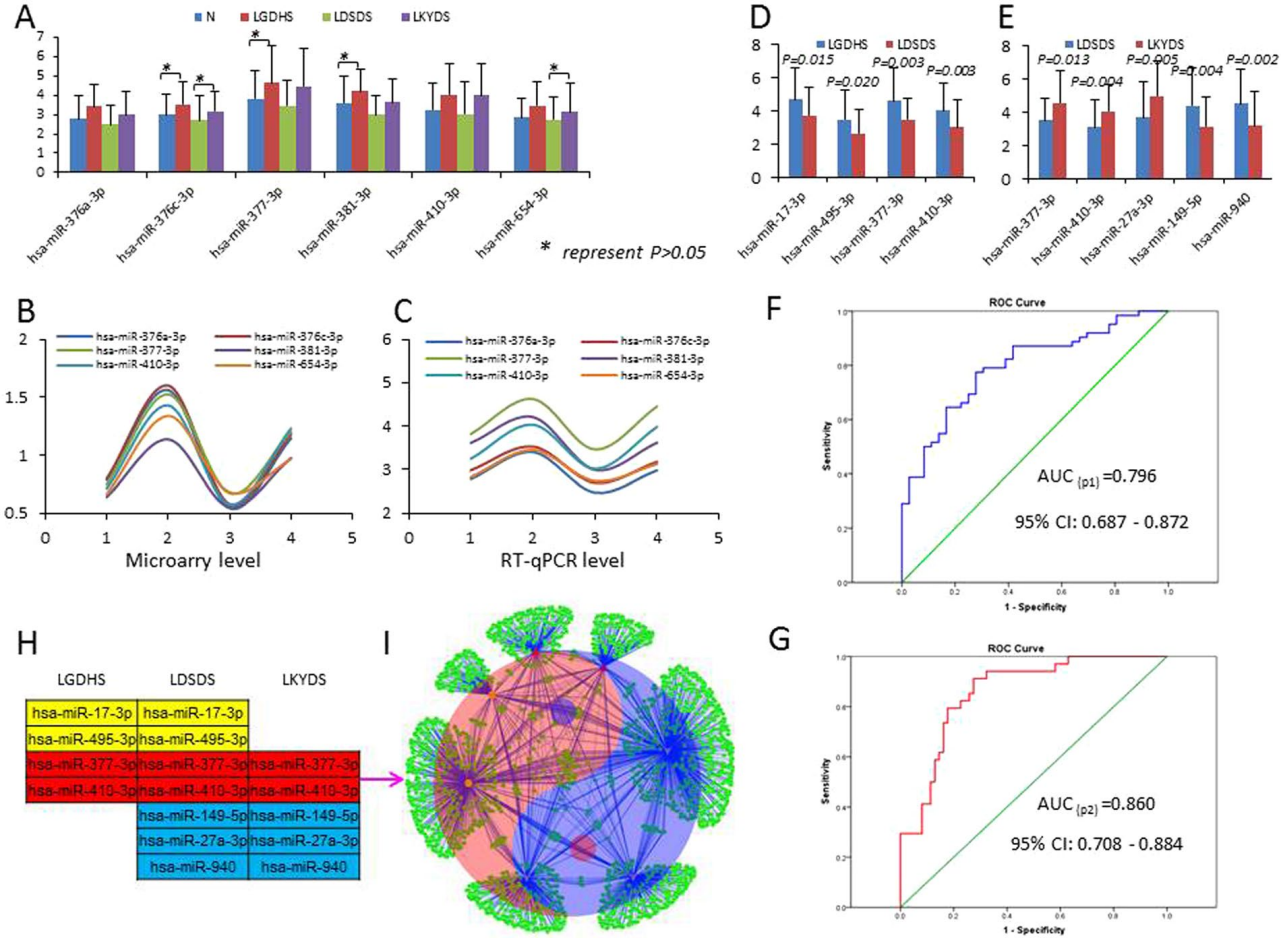
The co-expressed miRNAs and potential kernel miRNAs were validated by RT-qPCR in HBC TCM syndromes development. (**A**) Expression levels of 6 co-expressed miRNAs in HBC TCM syndromes development process. Illustrated P-values are based on pair-wise comparisons by Mann-Whitney U tests. The result shown that many miRNAs have statistical significance difference (P < 0.05), while hsa-miR-376c-3p, -377-3p and -381-3p in Normal/LGDHS, and hsa-miR-376c-3p and -654-3p in LKYDS/LDSDS (P > 0.05) are insignificant. The co-expressed miRNA expressed tendency of microarray (**B**) and RT-qPCR levels (**C**) are coherent in the HBC TCM syndromes development process. (**D**) Expression levels of 4 kernel miRNAs in LGHDS develop to LDSDS process. (**E**) Expression levels of 5 kernel miRNAs in LDSDS develop to LKYDS process. (**F**) ROC curve of p1 miRNA panel generated using 4 miRNA expression data and the AUC was 0.796. (**G**) ROC curve of p2 miRNA panel generated using 5 miRNA expression data and the AUC was 0.860. (**H**) The potential kernel miRNAs distribution in differential evolved stages of HBC TCM syndromes. (**I**) Using the potential kernel miRNAs to constructed the miRNA-target network, the topological profile shows a “Tai-Chi Diagram” of Chinese Traditional Medicine, 4 miRNAs located with “Yang” map and represents LGDHS) develop to LDSDS), 5 miRNAs located with “Yin” map and represents LDSDS develop to LKYDS, and the whole Tai-Chi Diagram represents the LDSDS in HBC TCM syndromes.

The RT-qPCR was performed to measure the expression levels of 6 co-expressed miRNAs in LGDHS-LDSDS-LKYDS process. At the transcriptional levels, most co-expressed miRNAs expression levels have statistically significance in whole evolutionary process of TCM syndromes, expect hsa-miR-376c-3p, -377-3p and -381-3p in Normal/LGDHS, and hsa-miR-376c-3p and -654-3p in LKYDS/LDSDS (P > 0.05)(Fig. 5A). Based on the RT-qPCR and microarray data, the expression tendency of co-expressed miRNAs are coherent in the evolutionary process of HBC TCM syndromes (Fig. 5B,C). This result suggests that the dynamical expression levels of co-expressed miRNAs might benefit to evaluate the development tendency of TCM syndromes in HBC progression.

To explore whether the kernel miRNAs play important roles in each evolution stage of HBC TCM syndromes, the selected kernel miRNAs also were validated (Fig. 5D,E). The statistical significance was noted for 4 miRNAs in LGDHS develop to LDSDS process (hsa-miR-17-3p, -377-3p, -410-3p, and 495), and 5 miRNAs in LDSDS develop to LKYDS process (hsa-miR-377-3p, -410-3p, -149-5p, 27a-3p, and -940). The results indicate that the biological functions of these miRNAs might highly correlate to the HBC TCM syndrome development.

### Identifying the excellent miRNA panel

Using RT-qPCR data, a Stepwise Logistic Regression Model was designed to calculate the optimal combination from these kernel miRNAs. Consequently, an excellent miRNA panel with hsa-miR-17-3p, -377-3p, -410-3p, and -495 were identified to evaluate the process of Excessive (LGDHS) develop to Excessive-deficient syndrome (LDSDS), and hsa-miR-377-3p, -410-3p, -27a-3p, 149-5p and 940 were identified evaluate the Excessive-deficient (LDSDS) evolved to Deficient syndrome (LKYDS) process. The logit model were (p1 = miRNAs) = 2.485-0.035 * hsa-miR-17-3p - 0.076 * hsa-miR-495-3p- 0.070 * hsa-miR-377-3p- 0.062 * hsa-miR-410-3p,and (p2 = miRNAs) = 1.117 + 0.072 * hsa-miR-377-3p + 0.059 * hsa-miR-410-3p- 0.051 * hsa-miR-149-5p + 0.051 * hsa-miR-27a-3p – 0.062 * has-miR-940, which were used to construct the ROC-curve. The AUC for the test were p1 = 0.796 (95% CI: 0.687 to 0.872, Fig. 5F) and p2 = 0.860 (95% CI: 0.708 to 0.884, Fig. 5G). This result suggested that multiple miRNAs can combined an excellent miRNA panel, and benefit to improve the diagnostic accuracy to evaluate the TCM syndrome development. Furthermore, it also implicating the differential expressed miRNAs might act as potential biomarkers for TCM syndromes classification in HBC progression.

To understand the potential functions of these kernel miRNAs holistically, a new miRNA-target network was constructed. At topological profile, we noticed that the network structure was more likely “Tai-Chi Diagram” of Chinese Traditional Medicine (Fig. 5H,I). In Tai-Chi Diagram, 4 miRNAs located with “Yang” map and represent LGDHS develop to LDSDS, whereas, 5 miRNAs belong to “Yin” map and represent LDSDS evolved to LKYDS, interestingly, the holistic Tai-Chi Diagram represents the LDSDS in TCM syndrome evolutionary process. This phenomenon suggests that the Excessive-deficient syndrome (LDSDS) is very important and might act as a critical point in the evolution process of HBC TCM syndrome.

## Discussion

As a class of regulator, miRNA plays a critical functional role in gene regulated progression^14^. In this study, we mainly focus on the miRNAs expression levels in the evolution process of HBC TCM syndromes, which was from Excessive (LGDHS) to Deficient (LKYDS) via Excessive-Deficient syndrome (LDSDS). The miRpath (v3.0) analysis shows Prion diseases, Signaling pathways regulating pluripotency of stem cells, and TGF-beta signaling pathway are highly correlated with the LGDHS, LDSDS, and LKYDS of HBC progression. Importantly, act as a central regulator, TGF-beta be involved in many disease progression in chronic liver disease, which from initial liver injury through inflammation/fibrosis to cirrhosis or hepatocellular carcinoma^15^. TGF-beta overexpression also was correlated with tumor progression, metastasis, angiogenesis and poor prognostic outcome^16–19^. It suggests that these pathways are important for understanding the mechanisms of HBC TCM syndromes development.

In the evolution process, six co-expressed miRNAs were identified among the LGDHS, LDSDS and LKYDS, which might essential links for HBC TCM syndrome development. The RT-qPCR demonstrates the expression tendencies of these co-expressed miRNAs are coherent with the microarray levels in the process of LGDHS develop to LKYDS. GO terms and KEGG pathways show that the target genes mainly associated with Binding, Transcription, Activity, Nucleoplasm, Prostate cancer, Non-small cell lung cancer, Glioma, Chronic myeloid leukemia, p53 signaling pathway and PI3K-Akt signaling pathway. The p53 signaling pathway was considered as highly correlated with the pathogenesis of numerous cancer^20^. In hepatitis B caused hepatocellular carcinoma (HCC), the transcriptional activity of p53 was impaired by interacting with NUMB and consequently HCC development^21^. Importantly, the serum p53 was increased in HBV-related cirrhosis patients^22^, and the serum p53 protein expression were more pronounced in patients with liver cirrhosis more than liver fibrosis^23^, furthermore, p53 also can act as potential biomarker for liver cirrhosis and HCC diagnosis^24^. This phenomenon suggests that the co-expressed miRNAs might regulate p53 signaling pathway related genes, and then to regulate the TCM syndromes development in whole HBC progression.

Furthermore, we found that 4 kernel miRNAs were associated with Excessive (LGDHS) develop to Excessive-Deficient (LDSDS), and 5 kernel miRNAs were correlated with Excessive-Deficient (LDSDS) develop to Deficient syndrome (LKYDS) based on RT-qPCR data. Using a Stepwise Logistic Regression Model, p1 miRNA panel (hsa-miR-17-3p, -377-3p, -410-3p and -495) and p2 miRNA panel (hsa-miR-377-3p, -410-3p, -27a-3p, 149-5p and 940) was identified, respectively, which was clearly improve the diagnostic accuracy of TCM syndrome in HBC progression. It suggests that these kernel miRNAs might act as useful tools to evaluate the evolution levels of HBC TCM syndromes. Interestingly, hsa-miR-377-3p and -410-3p not only overlapped between p1 and p2 miRNA panels, but also co-expressed in the whole evolutionary process of HBC TCM syndromes.

In p1 miRNA panel, all miRNAs are down expressed when LGDHS develop to LDSDS. Hsa-miR-377 was involved in the regulation of endogenous cell growth and associated with the problem pregnancies^25^. In nucleus pulposus cells, has-miR-377-3p was correlated with PKCε activation and might lead to ADAMTS5 long-term down-regulation^26^. Has-miR-377-3p also was demonstrated have tumor suppressive role and inhibited tumormetastasis by targeting E2F3 in NSCLC cell^27^. Has-miR-410 is a key regulatory factor and regulating the expression of IL-10 by targeting STAT3 in pathogenesis of Systemic lupus erythematosus (SLE)^28^. In liver and colorectal tumors, has-miR-410 have oncogenic properties and up-regulated by regulating FHL1^29^. Has-miR-410 also acts as a tumor suppressor gene in some malignancies, such as gastric cancer^30^, gliomas^31^, and pancreatic cancer^32^. Hsa-miR-17-3p was involved in cell cycle regulation and overexpressed in various types of human tumors^33^. In synovial sarcoma, hsa-miR-17-3p act as an oncogene and promote the tumor growth by directly inhibiting p21 expression^34^. Importantly, hsa-miR-495 could be a potential therapeutics in early tumorigenic progression, which was recover RUNX3 expression under hypoxic conditions^35^. In breast cancer, hsa-miR-495 can facilitate tumor progression through the repression of JAM-A^36^, especially, it can inhibit the G1-S phase transition and suppress proliferation and tumorigenicity^37^. Furthermore, hsa-miR-495 also was considered as a tumor suppressor and associated with many tumors regulation^38–42^.

In p2 miRNA panel, hsa-miR-377-3p, -410-3p and -27a-3p are up expressed, whereas, 149-5p and 940 are down expressed when LDSDS develop to LKYDS. Has-miR-27a-3p was reported as a candidate biomarker for Alzheimer disease (AD)^43^, and combines miR-23a to target SMAD5 and regulate cell apoptosis by the FasL-Fas pathway in human granulose^44^. In human livers, has-miR-27a can regulate the CYP3A4 gene expression^45^, and act as pharmacologically relevant modulators in liver Dihydropyrimidine dehydrogenase (DPD) function^46^. Has-miR149-5p was associated with cellular migration, proliferation and apoptosis in renal cell carcinoma^47^, act as potential prognosis of glioma^48^, mediate the crosstalk between tumor cells and cancer-associated fibroblasts in gastric cancer^49^, and increase hepatocellular carcinoma (HCC) risk^50–52^. The clinical evidence indicates that miR-149 is a potential prognostic biomarker of HCC, which was act as a tumor suppressive miRNA to inhibit tumorigenesis and repress metastasis of HCC^53, 54^. In HCC tissues and cell lines, has-miR-940 was remarkably decreased, and the lower expression level can promote cellular proliferation by targeting Estrogen-related receptor gamma (ESRRG)^55^. Importantly, the decreased has-miR-940 was contributed to poor overall survival of HCC, because it can decrease HCC invasion and migration by down-regulated CXCR2 expression^56^. Furthermore, has-miR-940 was consistently down-regulated in breast cancer tissues^57^, it can play oncogenic role in gastric cancer^58^, activates the Wnt/beta-catenin signaling activation by targeting GSK3β and sFRP1 in pancreatic carcinoma^59^, and act as a diagnostic and prognostic tool for prostate cancer^60^.

Compare the biological functions of these miRNAs, mainly of them were focus on tumor suppressive or acted as prognostic biomarkers for human tumors. Importantly, hsa-miR-149-5p and 940 of p2 miRNA panel were highly correlated with tumorigenesis and metastasis of HCC. The p2 miRNA panel was associated with the evolution process of Excessive-Deficient (LDSDS) to Deficient syndrome (LKYDS), and it suggested that the stage of LDSDS evolved to LKYDS was more dangerous and increased hepatocellular carcinoma (HCC) risk in HBC progression.

**Figure 6:**
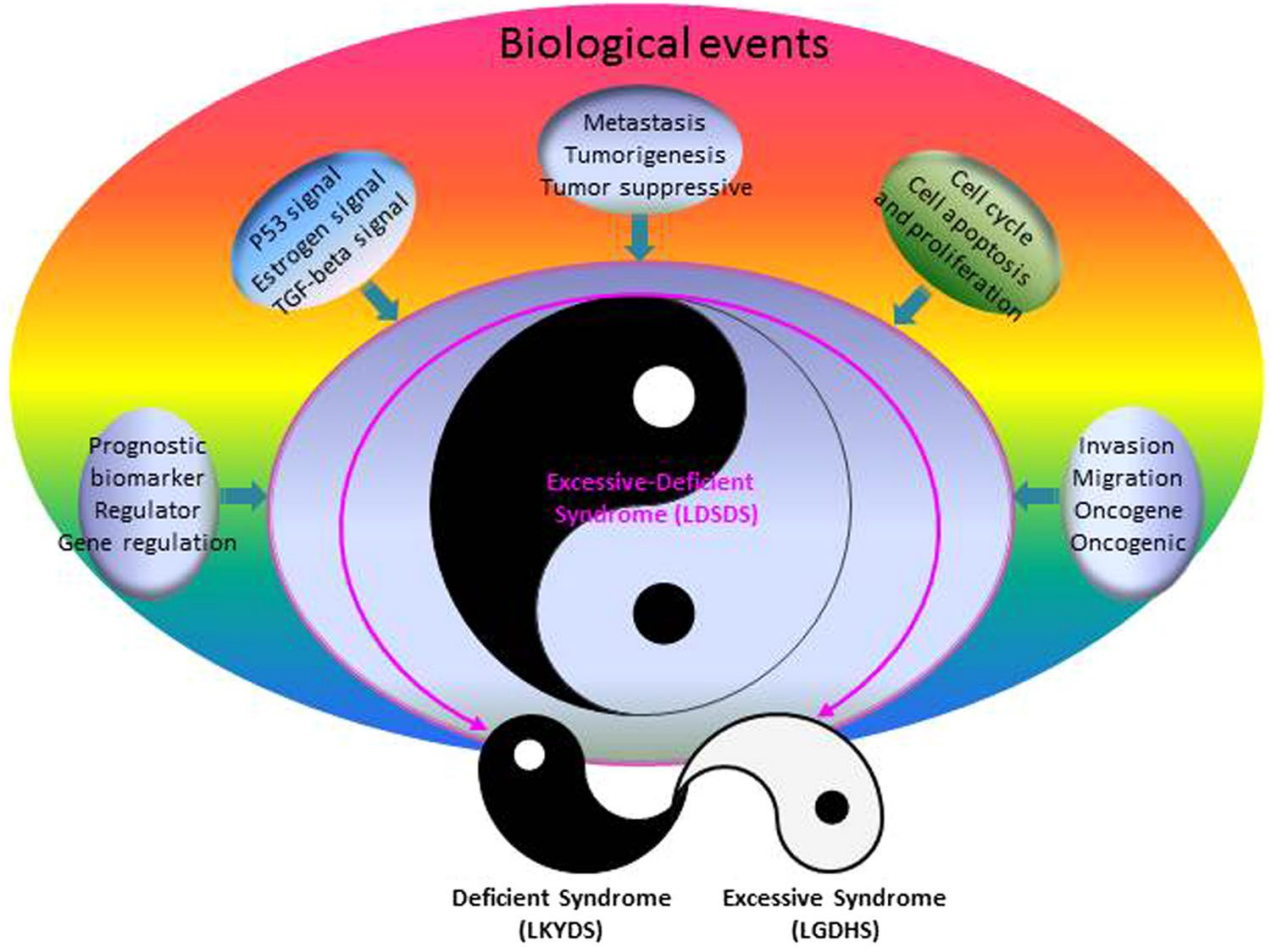
The illustration of LDSDS acts as critical point in the evolutionary process of HBC TCM syndromes, and a lot of biological events have a heavily concentrated in this stage. These biological events may determine the “right” or “left” evolutionary directions in the HBC TCM syndrome. In this model, the “right” evolved route represents LDSDS develop to LGDHS and the “left” evolved route represents LDSDS develop to LKYDS. Generally, the prognosis of Excessive (LGDHS) is better than that of Excessive-deficient syndrome (LDSDS) and Deficient syndrome (LKYDS) in HBC progression.

The miRNA-target networks show that the structure of LDSDS was most complicated in the evolutionary process of HBC TCM syndromes. Subsequently, the co-network was knockout from LGDHS, LDSDS and LKYDS related miRNA-target networks, respectively. The robustness levels show the topological profile of LDSDS network is stronger than others, and suggests the LDSDS is an important stage for HBC TCM syndrome development. Furthermore, a new miRNA-target network was rebuilt using p1 and p2 miRNA panel, and interestingly, a Tai-Chi Diagram appeared on the topological profile of network. In the Diagram, Yang map represents Excessive syndrome (LGDHS), Yin map represents Deficient syndrome (LKYDS), and the Yin-Yang map (whole diagram) represents Excessive-Deficient syndrome (LDSDS) of HBC progression. At previous, Yin-Yang background was used to describe the Yin-Yang theory of TCM^61^ and possible relationship between Cold-Hot ZHENG networks in Neuro-Endocrine-Immune (NEI) system^62^. Here, we infer that the Excessive-deficient syndrome (LDSDS) is an important critical functional point in the evolutionary routes of HBC TCM syndromes. A large number of biological events may be heavily concentrated in LDSDS stage, such as cell cycle, cell apoptosis and proliferation, tumorigenesis, tumor invasion, migration and metastasis, moreover, many regulation events like as p53 signaling pathway and TGF-beta signaling pathway also were occurred in this stage (Fig. 6). Obviously, these biological events might playing important roles in the LDSDS stage, and may determine the “right” or “left” evolved routes of TCM syndromes in HBC progression, especially, a mutual transformation between Excessive (LGDHS) and Deficient syndrome (LKYDS) may occur in given changes. Generally, the prognosis of Excessive (LGDHS) is better than that of Excessive-deficient syndrome (LDSDS) and Deficient syndrome (LKYDS) in HBC progression. If these biological events can effectively regulated and/or controlled, the LDSDS may developed to “right” direction of TCM syndromes (such as LGDHS), at least, it can delay the evolution process of LDSDS to LKYDS and slows down the poor prognosis in HBC progression.

In conclusion, we have identified a panel miRNAs to evaluate the development process of HBC TCM syndromes. Further analysis of the expression profiles of kernel miRNAs in different stages revealed that these miRNAs were distinctly expressed in certain stages of HBC TCM syndromes, which were significantly correlated with indicators for HBC TCM syndromes evolution. The topological profile of networks shows that the Excessive-Deficient syndrome (LDSDS) was more complicated than Excessive (LGDHS) and Deficient syndrome (LKYDS). All these findings would benefit to understand the expression profiles of miRNAs in different stages might act as novel diagnostic tools for TCM syndromes classification, and furthermore, the Excessive-Deficient syndrome (LDSDS) is a critical point of TCM syndromes when Excessive (LGDHS) develop to Deficient syndrome (LKYDS), which acts as a mediated process and determined the evolution direction of HBC TCM syndrome.

## Methods

### Clinical specimens

In this study, 162 clinical serum samples were collected from Shanghai Shuguang Hospital, which was including LGDHS (n = 36), LDSDS (n = 62), LKYDS (n = 34), and Normal controls (n = 30). The diagnostic criteria of western medicine for HBC followed the guidelines that defined by the Chinese Society of Hepatology and Chinese Society of Infectious Diseases in 2005^63^. The TCM syndrome system for HBC applied by the 3 senior TCM doctors of each diagnosis was accepted according to the standards of TCM differential syndromes of viral hepatitis that defined by the Internal Medicine Hepatopathy Committee of Chinese Traditional Medicine Association in 1991^64^. This research project approved with the local ethics committee of Shanghai University of TCM, and we confirm that a statement: (i) identifying the institutional and/or licensing committee approving the experiments, including any relevant details; (ii) confirming that all experiments were performed in accordance with relevant guidelines and regulations. All patients were informed consent for this study, and patients’ names and other HIPAA identifiers were removed from all sections of the manuscript, including supplementary information. Furthermore, to investigate the miRNA profiles, we selected 9 HBC serums (LGDHS n = 3, LDSDS n = 3, and LKYDS n = 3) and 7 Normal donors to miRNA microarray analysis. The differentiation of TCM syndromes in HBC patients was shown in Table 1, and the ALT, AST and GGT have not statistically significant differences among the three TCM stages.

**Table 1:** Clinical data of subjects^a^.

Characteristics	LGDHS (mean ± SD)	LDSDS (mean ± SD)	LKYDS (mean ± SD)
Age (years)	49.3 ± 8.5	50.1 ± 8.7	49.6 ± 8.3
Gender			
Male (n)	16	36	18
Female (n)	20	26	16
Total	36	62	34
HBV History (years)	14.1 ± 10.3	14.2 ± 10.4	14.4 ± 10.7
ALT (U/L)	48.83 ± 41.43	45.58 ± 33.61	48.79 ± 46.09
AST(U/L)	59.62 ± 45.85	58.81 ± 43.55	60.47 ± 47.44
GGT(U/L)	58.51 ± 53.65	63.78 ± 60.24	60.12 ± 64.34

^a^Note: SD, standard deviation; Alanine aminotransferase, ALT; Aspartate transaminase, AST; Glutamyl transpeptidase, GGT.

### miRNA profiles detection and analysis

The miRNA profiles were generated using Agilent Human miRNA microarray V3 (Agilent Technologies Inc, Santa Clara). All raw data were transformed to log2 and normalized each expression by zero mean and unit sample variance.

Using R package, a random variance model was designed to indentify the differential expressed (DE) miRNAs among the three TCM syndromes of HBC, such as LGDHS/Normal, LDSDS/Normal, LKYDS/Normal, LDSDS/LGDHS and LKYDS/LDSDS, where the fold-change >1.5 and P < 0.05 were considered to be significant. Heat-map and hierarchical analysis were performed using Cluster 3.0 and Java TreeView programs. Furthermore, the miRPath (v3.0)^11^ were performed for analysis the classified miRNAs related pathways (*P* < 0.001), which were based on the TarBase database (v7.0)^65^.

### miRNA target genes prediction

The miRNA target genes were predicted using three database involve TarBase (v7.0), miRecords^66^ and miRTarBase^67^, which contained the largest collection of manually curate experimentally data. Furthermore, miRanda, miRDB, miRWalk, and RNAhybrid programs were used to predict the non-experimental targets, where the P < 0.001 were considered to be significant. The predicted target genes were analyzed using DAVID online^68, 69^, significance analysis was defined as P value adjusted by False Discovery Rate (FDR), and gene sets containing less than 5 genes overlapping were removed. In this study, Gene ontology (GO), pathway and disease term with an FDR-adjusted P-value of less than 0.05 were retained.

### miRNA-target network construction

The differential expressed (DE) miRNAs and predicted targets were combined and to construct miRNA-target network. Being built network, miRNAs were weighted by the fold change (|log2|), target genes were weighted based on the distribution of degree. Subsequently, all nodes were ranked according to their weights and tested the similarity, then, the obtained nodes were used to remap the network. In the network, the node represents miRNA or target, the edge represents the connection strength.

In this study, the co-network was knockout from raw network, and the robustness (R) level was used to evaluate the stability of network. The formula as follows,

$${\rm{R}}=\frac{{\rm{C}}}{({\rm{N}}-{\rm{N}}{\rm{\tau }})}$$

where the numerator C is the maximum connected component after network knockout, N is the raw network data, and Nτ is the number of knockout nodes. Furthermore, the consecutive parameters of network were generated, such as Betweenness Centrality (BC), Closeness Centrality (CC) and Degree (De). In this work, the kernel node of network was defined as BC ≥ Avg (BC), CC ≥ Avg (CC) and De ≥ Avg (De). The determinate miRNAs were considered as kernel miRNAs and play important roles in differential HBC TCM syndromes.

### Quantification of co-expressed miRNAs and kernel miRNAs

The RT-qPCR was used to identify the co-expressed miRNAs and kernel miRNAs in 162 serum samples (LGDHS n = 36, LDSDS n = 62 and LKYDS n = 34, Normal controls n = 30). The quantification of miRNA was performed with SYBR Green PCR Master Mixture (TOYOBO, LTD, Japan) according to the manufacturer’s instructions using a Rotor-Gene 6000 Real-time PCR machine (Corbett Life Science, Sydney, Australia). The specificity of each PCR product was validated by melting curve at the end of PCR cycles. All miRNAs were validated in triplicate, the Ct was considered as the number of cycle requirement and for the fluorescent signal to reach the threshold. The levels of miRNAs were calculated using 2^ΔCt^, where ΔCt = Ct of internal reference -Ct of target miRNA. The differences in miRNAs expression levels between groups were compared using the Student’s t-test and P-value < 0.05 was considered have statistically significant difference.

### Experimental data analysis

Using RT-qPCR data, a stepwise logistic regression model was performed to screen diagnostic miRNA panel, which was considered as potential markers in the evolutionary process of HBC TCM syndromes. The predicted probability of being diagnosed with TCM syndromes evolution was used as surrogate marker to construct a receiver operating characteristic (ROC) curve. The area under the ROC-curve (AUC) represents an accuracy index for evaluating the diagnostic performance of the selected miRNA panel. The two-tailed test was used and P < 0.05 was considered have statistically significant.

## Electronic supplementary material

Supplementary Table 1
