# Author Correction: MiRNA-target network analysis identifies potential biomarkers for Traditional Chinese Medicine (TCM) syndrome development evaluation in hepatitis B caused liver cirrhosis

**DOI:** 10.1038/s41598-020-63940-6

**Published:** 2020-04-24

**Authors:** Yamin Liu, Mei Wang, Yunquan Luo, Jian Chen, Yiyu Lu, Yulin Shi, Chenchen Tang, Qianmei Zhou, Hui Zhang, Yuanjia Hu, Shibing Su, Qilong Chen

**Affiliations:** 1 Research Center for TCM Complexity System, Shanghai University of TCM, Shanghai, 201203 China; 2 School of Clinical Medicine, Shanghai University of Medicine & Health Sciences, Shanghai, 201203 China; 3 Shu Guang Hospital Affiliated to Shanghai University of TCM, Shanghai, 201203 China; 4 State Key Laboratory of Quality Research in Chinese Medicine, Institute of Chinese Medical Sciences, University of Macau, Macau, China

Correction to: *Scientific Reports* 10.1038/s41598-017-11351-5, published online 08 September 2017

The original version of this Article contained an error in the name of the author Jian Chen, which was incorrectly given as ChenJian Chen. In addition, in the Author Contribution statement and the supplementary material accompanying this Article, the first and last names of all the authors were inadvertently switched. The Author Contribution statement now reads:

S.B.S. and Q.L.C. designed the research project. Y.M.L., M.W., Y.Q.L., J.C. and H.Z. performed all the experiments. Y.Y.L., Y.L.S., C.C.T. and Q.M.Z. conducted the bioinformatics analysis. Y.J.H. helped with the results analysis. Y.M.L. and Q.L.C. wrote the manuscript. All authors reviewed the manuscript.

These errors have now been corrected in the HTML and PDF version of the Article, and in the accompanying supplementary material.

