# Supplementary material for: MiRNA-target network analysis identifies potential biomarkers for Traditional Chinese Medicine (TCM) syndrome development evaluation in hepatitis B caused liver cirrhosis
Source: Sci Rep. 2017 Sep 8;7:11054. doi: 10.1038/s41598-017-11351-5 (PMC5591282; doi:10.1038/s41598-017-11351-5)
Supplement: Supplementary file 1 — Supplementary Table 1 [file 41598_2017_11351_MOESM1_ESM.doc]

MiRNA-target network analysis identifies potential biomarkers for Traditional Chinese Medicine (TCM) syndrome development evaluation in hepatitis B caused liver cirrhosis 
 
Yamin Liu1#, Mei Wang2#, Yunquan Luo3#, Jian Chen1#, Yiyu Lu1, Yulin Shi1, Chenchen Tang1 , Qianmei Zhou1 , Hui Zhang1 , Yuanjia Hu4 , Shibing Su1*, Qilong Chen1* 
 
1 Research Center for TCM Complexity System, Shanghai University of TCM, Shanghai 201203, China 
2 School of Clinical Medicine, Shanghai University of Medicine & Health Sciences, Shanghai 201203, China 
3 ShuguangHospital Affiliated to Shanghai University of TCM, Shanghai 201203, China 
4 State Key Laboratory of Quality Research in Chinese Medicine, Institute of Chinese Medical Sciences, University of Macau, Macau, China 
# These authors are contribute for this paper 
* Corresponding author: Chen Qilong and SuShibing, Tel: +86-21-51322755, Fax: +86-21-52323013, E-mail:cqlw1975@126.com, Address: No.1200 Cailun Road, Shanghai, 201203, China.  
  

Supplementary Table 1. The kernel miRNAs selected from LGDHS, LDSDS and LKYDS miRNA-target networks 
  	symbol	Closeness	Betweeness	Degree	
LGDHS	hsa-miR-136-5p 	0.346859 	0.093694 	181 	
	hsa-miR-17-3p 	0.388499 	0.336635 	620 	
	hsa-miR-215-5p 	0.404247 	0.420668 	778 	
	hsa-miR-377-3p 	0.356908 	0.150477 	286 	
	hsa-miR-410-3p 	0.353381 	0.118466 	245 	
	hsa-miR-495-3p 	0.350844 	0.104005 	212 	
	hsa-miR-574-5p 	0.367926 	0.21826 	406 	
LDSDS	hsa-miR-101-3p 	0.362824 	0.100228 	785 	
	hsa-miR-1228-3p 	0.338479 	0.026133 	221 	
	hsa-miR-144-3p 	0.34314 	0.015438 	266 	
	hsa-miR-149-5p 	0.353466 	0.085555 	551 	
	hsa-miR-17-3p 	0.356531 	0.085604 	620 	
	hsa-miR-19b-3p 	0.375328 	0.174428 	1087 	
	hsa-miR-22-5p 	0.348854 	0.052379 	436 	
	hsa-miR-27a-3p 	0.385761 	0.156364 	1324 	
	hsa-miR-27b-3p 	0.377309 	0.106626 	1133 	
	hsa-miR-374a-5p 	0.358396 	0.088534 	673 	
	hsa-miR-377-3p 	0.343858 	0.042507 	286 	
	hsa-miR-410-3p 	0.341785 	0.026839 	245 	
	hsa-miR-495-3p 	0.340441 	0.021126 	212 	
	hsa-miR-766-3p 	0.364634 	0.131349 	825 	
	hsa-miR-877-3p 	0.362025 	0.125122 	775 	
	hsa-miR-92b-3p 	0.36581 	0.132312 	860 	
	hsa-miR-939-5p 	0.344074 	0.037255 	299 	
	hsa-miR-940 	0.360953 	0.119611 	739 	
	hsa-miR-1267 	0.338867 	0.068334 	115 	
LKYDS	hsa-miR-1470 	0.341151 	0.115104 	181 	
	hsa-miR-149-5p 	0.371278 	0.249451 	381 	
	hsa-miR-27a-3p 	0.371169 	0.25298 	391 	
	hsa-miR-377-3p 	0.35673 	0.162668 	253 	
	hsa-miR-381-3p 	0.34328 	0.076311 	133 	
	hsa-miR-410-3p 	0.354036 	0.14963 	245 	
	hsa-miR-940 	0.413359 	0.471202 	739 	
 
